# White rot fungal impact on the evolution of simple phenols during decay of silver fir wood by UHPLC‐HQOMS

**DOI:** 10.1002/pca.3077

**Published:** 2021-07-28

**Authors:** Stefania Di Lella, Nicola La Porta, Roberto Tognetti, Fabio Lombardi, Tiziana Nardin, Roberto Larcher

**Affiliations:** ^1^ Department of Biosciences and Territory University of Molise Pesche Italy; ^2^ Research and Innovation Centre Fondazione Edmund Mach San Michele all'Adige Italy; ^3^ Department of Agricultural, Environmental and Food Sciences University of Molise Campobasso Italy; ^4^ The EFI Project Centre on Mountain Forests (MOUNTFOR) Edmund Mach Foundation Trento Italy; ^5^ Department of Agraria University Mediterranea of Reggio Calabria Reggio Calabria Italy; ^6^ Technology Transfer Centre Fondazione Edmund Mach San Michele all'Adige Italy

**Keywords:** *Armillaria ostoyae*, *Heterobasidion abietinum*, lignin decay, phenolic compounds, silver fir, white rot fungi

## Abstract

**Introduction:**

Silver fir ( *Abies alba* Mill.) is one of the most valuable conifer wood species in Europe. Among the main opportunistic pathogens that cause root and butt rot on silver fir are *Armillaria ostoyae* and *Heterobasidion abietinum*. Due to the different enzymatic pools of these wood‐decay fungi, different strategies in metabolizing the phenols were available.

**Objective:**

This work explores the changes in phenolic compounds during silver fir wood degradation.

**Methodology:**

Phenols were analyzed before and after fungus inoculation in silver fir macerated wood after 2, 4 and 6 months. All samples were analyzed using high‐performance liquid chromatography coupled to a hybrid quadrupole‐orbitrap mass spectrometer.

**Results:**

Thirteen compounds, including simple phenols, alkylphenyl alcohols, hydroxybenzoketones, hydroxycinnamaldehydes, hydroxybenzaldehydes, hydroxyphenylacetic acids, hydroxycinnamic acids, hydroxybenzoic acids and hydroxycoumarins, were detected. Pyrocatechol, coniferyl alcohol, acetovanillone, vanillin, benzoic acid, 4‐hydroxybenzoic acid and vanillic acid contents decreased during the degradation process. Methyl vanillate, ferulic acid and *p*‐coumaric were initially produced and then degraded. Scopoletin was accumulated. Pyrocatechol, acetovanillone and methyl vanillate were found for the first time in both degrading and non‐degrading wood of silver fir.

**Conclusions:**

Despite differences in the enzymatic pool, both fungi caused a significant decrease in the amounts of phenolic compounds with the accumulation of the only scopoletin. Principal component analysis revealed an initial differentiation between the degradation activity of the two fungal species during degradation, but similar phenolic contents at the end of wood degradation.

## Abstract 2

Phenols were evaluated during silver fir degradation after inoculation with fungi.

## 1 INTRODUCTION

Phenolic compounds are commonly produced as secondary metabolites from plant and fungal species. In plants they constitute one of the most common and widespread groups of substances which arise biogenetically from the pentose phosphate, shikimate and phenylpropanoid pathways. The plant‐associated fungi have symbiotically adopted these pathways into their metabolic cycle and mimic the plants by producing phenols.^1^ Plants need phenolic compounds for antifungal activity and resistance to pathogen growth; moreover, both plants and fungi use them for pigmentation, reproduction and many other functions.^2^

The fungi *Armillaria* spp. and *Heterobasidion* spp. have dual life strategies, being necrotrophic on living trees and subsequently saprotrophic on dead wood. As both fungi are considered white rot fungi, they have a versatile machinery of enzymes to attack directly the lignin barrier. The two fungal genera differ also significantly in symptomatology.^3^ Recently, the genomes of a European and a North American *Armillaria ostoyae* strain were published^4^ while the *Heterobasidion abietinum* sequence is not described yet. Interestingly, in comparison with other white rot fungi, *Armillaria* shows an underrepresentation of ligninolytic gene families and an overrepresentation of pectinolytic gene families.^4^ Accordingly, recent studies considered *Armillaria* spp. as white rot fungus, based on the presence of genes encoding lignin‐decaying enzymes in their genomes.^4^, ^5^, ^6^, ^7^ However, previous studies have also shown that *Armillaria* species primarily decay the cellulose, hemicellulose and pectin components of the plant cell wall, and leave lignin unattacked during early stages of decay.^8^ They have been discussed as white rot species, though their response to wood deviates from that of typical white rotters. While we observed an upregulation of a diverse suite of plant cell wall‐degrading enzymes, unlike white rotters, they possess and express an atypical wood‐decay repertoire in which pectinases and expansins are enriched, whereas lignin‐decaying enzymes are generally downregulated.^9^ *Heterobasidion* spp. have more gene families typically involved in lignin degradation or modification, including laccases and peroxidase.

Results for wood block degradation correlated well with the ability of the *Heterobasidion* spp. to produce laccase in liquid and solid culture conditions, with *H. annosum ss*. producing ca 5–6 times more laccase than *H. parviporum*, indicating that great differences exist between *Heterobasidion* species' abilities to cause wood decay.^10^ The analysis of the *Heterobasidion irregulare* genome revealed a repertoire of genes encoding lignocellulose‐degrading enzymes, including 179 glycoside hydrolases (GHs), eight manganese peroxidases (MnPs) and 17 multicopper oxidases (MCOs).^11^ In a study on the degradation of pine wood during saprotrophic growth of *H. annosum ss*., the induction of many GHs, MCOs, five MnPs and one oxidoreductase was observed, being specific for wood degradation. A total of 31 predicted GH genes were found upregulated in heartwood, 20 in sapwood and 23 in bark compared to the control.^12^ In *Heterobasidion parviflorum*, a close relative of *H. abietinum*, it was proved that in addition to transcriptome variation, also variation in the methylome (DNA cytosine methylation) is an important epigenetic modification in the lifestyle transition of this fungus.^13^ After wounding and inoculation of the bark of Sitka spruce, different concentrations of cell wall‐bound phenolic compounds from the necrotrophic lifestyle of *H. annosum* were found, including unknown2, unknown3, coniferin, astringin, taxifolin, piceid and isorhapontin, whereas in sapwood the concentrations did not differ following treatment. These results indicate that bark of Sitka spruce has a stronger and earlier response to wounding and pathogen inoculation than sapwood.^14^

White rot fungi have been investigated extensively since the mid‐1980s for their bioremediation capacities;^15^ in fact, they are considered the only organisms able to completely decompose lignin into CO_2_ and water.^16^, ^17^, ^18^ Lignin, the second largest sink of fixed carbon, after cellulose,^19^ is a complex phenolic biopolymer that plays a central role in mechanical support of plant cell walls, water transport and pathogen resistance in plants.^20^ The lignin molecule can be composed of three different phenylpropane monomer units (monolignols), namely, *para*‐coumaryl alcohol, coniferyl alcohol and sinapyl alcohol, linked by ether and carbon–carbon type bonds.^21^ The composition and amount of this polymer vary depending on the different botanical groups (conifers vs. broadleaves), between different tree species and even between the different woody tissues of the same tree.^22^ Conifers (softwood) are known to contain high amounts of lignin consisting mainly of guaiacyl units (90%) derived from coniferyl alcohol; broadleaf trees (hardwoods) and herbaceous species contain similar amounts of both guaiacyl and syringyl units derived from coniferyl alcohol and sinapyl alcohol, respectively.^23^, ^24^ Moreover, softwood lignin is estimated to be comprised of about 19% to 26% of phenolic units, whereas hardwood lignin contains about 14 to 18% of phenolic units.^25^ Due to the chemically complex structure, lignin polymer is highly resistant to physical, chemical and biological degradation.^26^

In order to depolymerize and mineralize the complex lignin molecule, these fungi secrete various combinations of strong extracellular oxidative lignolytic enzymes known as “ligninases”.^27^ Ligninases include mainly lignin peroxidase, MnP and laccase.^28^, ^29^ In particular, lignin peroxidase directly attacks the non‐phenolic lignin model compounds, such as veratryl alcohol (VA), by producing intermediate radicals,^30^, ^31^ whereas Mn‐dependent peroxidase and laccase are able to oxidize the phenolic lignin units to phenoxy radicals, leading to the decomposition of the woody structures.^32^, ^33^ Different combinations of these enzymes produced by white rot fungi underlie different mechanisms of lignin degradation with the production of various phenolic compounds.^34^, ^35^, ^36^ For example, the degradation of guaiacyl‐β‐coniferyl ether in softwood lignin (e.g. pine and spruce lignins) leads to the formation of coniferyl alcohol, coniferylaldehyde, ferulic acid, several low‐molecular weight aromatic acids and aldehydes, including vanillin and vanillic acid.^37^, ^38^, ^39^, ^40^ Other common degradation products of hardwood are syringic acid, syringaldehyde, protocatechuic acid and gallic acid.^41^ Many studies have extensively investigated the role and activity of ligninolytic enzymes produced by white rot fungi, such as *Phanerochaete chrysosporium*, during lignin depolymerization.^42^, ^43^, ^44^, ^45^ Several studies have also described different strategies of fungi in metabolizing the phenolic compounds during wood degradation^24^, ^46^, ^47^; however, only a preliminary study has focused on silver fir (*Abies alba* Mill.) wood which evaluated the distribution and variation of extractable total phenols and tannins in the logs of four conifers after 1 year on the ground.^48^ Silver fir, one of the most valuable conifer wood species in Europe, is widely distributed in Central and Southern European forests and therefore of significant ecological and economic value.^49^ Among the main opportunistic pathogens that cause root and butt rot on silver fir are *A. ostoyae* ^50^ and *H. abietinum*,^51^, ^52^ which was previously called *Heterobasidion annosum* F‐group.^53^ The infections caused by these fungi can spread from tree to tree through root connections. When a host tree dies or is cut, both pathogenic fungi act as strong saprophytic organisms^54^, ^55^; consequently, these fungi are of major importance in the decay of living trees and of dead silver fir wood.

Various chromatographic techniques have been used to identify and quantify phenolic compounds of lignin, including gas chromatography (GC), liquid chromatography (LC), size exclusion chromatography (SEC), capillary electrophoresis (CE) and two‐dimensional chromatography.^56^ However, among these analytical techniques, the coupling of high‐performance LC and mass spectrometry (HPLC‐MS) proved to be a powerful technique for the analysis of low‐molecular weight compounds such as phenols with high selectivity and sensitivity.^57^, ^58^

Considering the already well‐described lignin degradation realized by rot fungi^59^ and also in vitro degradation,^60^, ^61^ this work aimed to study how the different enzymatic pools of the two rot fungi influenced the trend of phenolic compounds at different wood degradation periods (2, 4 and 6 months) on macerated silver fir wood. Phenolic profiles were explored before and after fungal inoculations in the laboratory using HPLC coupled to a hybrid quadrupole‐orbitrap mass spectrometer (LC‐Q‐Orbitrap).

## 2 EXPERIMENTAL PROCEDURES

### 2.1 Reagents and standards

LC‐MS grade acetonitrile (99.9%), LC‐MS grade methanol (99.9%) and MS grade formic acid (98%) were purchased from Fluka (St. Louis, MO, USA). Potato dextrose agar (PDA) was purchased from Oxoid (Hampshire, England). Deionized water used for the preparation of the sample and eluent solutions was obtained using an Arium®Pro Lab Water System (Sartorius AG, Goettingen, Germany). The phenol standards used for quantitative determination were grouped into 15 classes according to their chemical structure^62^ (Table 1). According to the solubility of the various phenols, six aqueous solutions with different percentages of methanol, ranging from 15% to 55%, were used in the preparation of the stock solutions (Table 1, MeOH %). Furthermore, phenols with the same *m*/*z* were dissolved in separate solutions, for a final of eight total mixtures (Table 1, I.D.). The stock solutions were then combined into a single intermediate solution (water–methanol mixture; 75:25 v/v) with a concentration of 10 mg L^−1^ for each phenol and freshly diluted to the desired concentration before each analysis. Stock solutions were stored at −4°C. Mass calibration solution (Pierce® ESI Negative Ion Calibration Solution) was purchased from Thermo Fisher Scientific Inc. (Waltham, MA, USA).

**TABLE 1:** Technical characteristics of phenolic analytical standards

Phenolic compounds			Stock solution	
	Purity	Supplier	I.D.	MeOH%
**Simple phenols**				
Phenol	≥99%	Sigma Aldrich	4	22
Pyrocatechol	≥99%	Sigma Aldrich	3	22
**Alkyphenols**				
4‐Ethylcatechol	≥98%	Sigma Aldrich	4	55
4‐Methylcatechol	≥98%	Fluka	5	22
4‐Vinylphenol	n.d.	Sigma Aldrich	5	55
*m*‐Cresol	≥98%	Sigma Aldrich	5	55
*o*‐Cresol	≥99%	Sigma Aldrich	5	35
*p*‐Cresol	≥99.9%	Sigma Aldrich	6	55
**Methoxy‐ and alkylmethoxyphenols**				
4‐Ethylguaiacol	≥98%	Sigma Aldrich	6	55
4‐Methylguaiacol	≥99%	Sigma Aldrich	6	55
4‐Vinylguaiacol	≥98%	Sigma Aldrich	6	55
Guaiacol	≥99%	Sigma Aldrich	6	55
**Dimethoxyphenol and alkylphenylmethoxy alcohols**				
4‐Methylsyringol	≥97%	Sigma Aldrich	7	55
Syringol	≥99%	Sigma Aldrich	7	35
**Alkylphenyl alcohols**				
Coniferyl alcohol	≥98%	Sigma Aldrich	3	22
Hydroxytyrosol	≥98%	Sigma Aldrich	7	22
Homovanillyl alcohol	≥99%	Sigma Aldrich	5	35
**Hydroxyphenylpropenes**				
Eugenol	≥99%	Fluka	7	55
Isoeugenol	≥98%	Sigma Aldrich	8	35
**Hydroxybenzoketones**				
Acetosyringone	≥97%	Sigma Aldrich	1	40
Acetovanillone	≥98%	Sigma Aldrich	3	40
Ethyl vanillate	n.d.	Sigma Aldrich	8	35
Isoacetosyringone	≥97%	Sigma Aldrich	3	40
Isoacetovanillone	≥97%	Sigma Aldrich	8	40
Isopropiovanillone	≥96%	Sigma Aldrich	7	40
Methyl vanillate	≥99%	Sigma Aldrich	2	40
**Hydroxybenzoether**				
Vanillyl ethyl ether	n.d.	Fluka	8	35
**Hydroxycinnamaldehydes**				
Coniferylaldehyde	≥98%	Sigma Aldrich	2	55
Sinapinaldehyde	≥98%	Sigma Aldrich	4	55
**Hydroxybenzaldehydes**				
Syringaldehyde	≥98%	Sigma Aldrich	2	40
Vanillin	≥99%	Sigma Aldrich	1	40
**Hydroxyphenylacetic acids**				
Homovanillic acid	≥98%	Sigma Aldrich	1	15
**Hydroxycinnamic acids**				
Caffeic acid	≥95%	Fluka	4	15
Ferulic acid	≥98%	Fluka	2	15
*p*‐Coumaric acid	≥98%	Sigma Aldrich	1	15
**Hydroxybenzoic acids**				
Benzoic acid	≥99.5%	Sigma Aldrich	3	15
Gallic acid	≥97.5%	Sigma Aldrich	4	15
Gentisic acid	≥98%	Fluka	4	15
4‐Hydroxybenzoic acid	≥99%	Fluka	1	15
Protocatechuic acid	≥97%	Fluka	2	15
Syringic acid	≥97%	Sigma Aldrich	3	15
Vanillic acid	≥97%	Fluka	3	15
**Hydroxycoumarins**				
Aesculetin	≥98%	Sigma Aldrich	8	30
Scopoletin	≥98.5%	Sigma Aldrich	1	30
**Flavanols**				
(−)‐Epicatechin	≥90%	Sigma Aldrich	7	30
(+)‐Catechin	≥98.5%	Fluka	8	30

n.d. = not detected; I.D. = identical dilution.

### 2.2 Evaluation of sample preparation

The extraction procedure is an important step for the quantification of wood extractives, such as phenolic compounds. The extraction yield of phenolics is affected by several factors, such as the solvents used with varying polarities, the sample‐to‐solvent ratio, extraction time, temperature and the characteristics of the sample.^63^ Studies have reported that different solvents, such as methanol, ethanol, acetone, ethyl acetate and their combinations with different proportions of water, were used for the extraction of phenolic compounds from wood and plant materials.^64^, ^65^ In the present study, the extraction of phenolic compounds from macerated silver fir wood containing the fungal inoculum was evaluated at two different temperatures (30°C and 80°C), using five different mixtures of methanol/water: 100% H_2_O, 100% MeOH and H_2_O/MeOH at 75:25 (v/v), 50:50 (v/v) and 25:75 (v/v). The different sample preparation tests were conducted on a sample of silver fir sapwood taken from a living tree located in the Trentino Region (Italy).

### 2.3 Fungal strains, culture conditions and sample preparation

Two different species of white rot fungi, *A. ostoyae* and *H. abietinum*, obtained from the fungal culture collection of the Pathology Lab of the Edmund Mach Foundation (TN), were used. In order to promote mycelium growth, both fungal species were axenically cultivated on 20 g L^−1^ of sterile PDA plates for about 20 days at room temperature. A total of 12 Petri dishes were prepared (six replicates for each fungal species). After fungal growth, the mycelium of *A. ostoyae* and *H. abietinum* was scratched off from the plates and cut into small pieces using a sterile scalpel blade and then inoculated axenically onto 3 g of sterile macerated silver fir wood in 18 sterile glass vials of 10 cm^3^ (Sartorius AG, Goettingen, Germany), in nine replicates per each fungal species, out of which three were harvested per time point. Macerated wood was prepared by milling sapwood of silver fir with an M20 mechanical mill (IKA–WERKE, Staufen, Germany) to obtain wood chips of about 3 mm in diameter. Silver fir sapwood was previously taken from a living tree located in the “Abeti Soprani” forest in the Molise Region (Italy) using a chainsaw. To promote fungal growth in macerated silver fir wood, 5 mL of sterile ultra‐pure water was added in each glass vial.^66^ In addition to the vials with fungal inoculum, three other vials were prepared as controls (t0), containing only sterile macerated wood, which were stored at −20°C. The inoculated vials were stored at room temperature and after two (t1), four (t2) and six (t3) months, six of them (three for each fungus) were put in the freezer to stop fungal activity and preserve the samples until analysis. Before the analysis of phenolic compounds, all samples were dried in the oven at 30°C for 1 week. After drying, 0.1 g of each sample was transferred into 2‐mL Eppendorf tubes and then extracted in 2 mL of water–methanol mixture (H_2_O/MeOH 75:25, v/v). The solutions were first homogenized for 2 min using an Ultrasonic processor (UP50H; 50 watts, 30 kHz; Hielscher Ultrasonics GmbH, Warthestraße, Germany) and were then shaken using a Multi Reax (Heidolph Instruments GmbH & Co. KG, Schwabach, Germany) for 15 min. The samples were centrifuged at 15,000 rpm and 10°C for 5 min and then suspended again with the Multi Reax; afterwards, they were left to rest for 1 hour. Finally, all samples were centrifuged for 5 min at 15,000 rpm, the supernatants were filtered with 0.45‐μm PTFE filter cartridges (Sartorius AG) into analytical 2‐mL vials and 10 μL per sample was injected.

### 2.4 LC‐HRMS analysis

The analysis of phenolic compounds was carried out according to the method described by Barnaba et al. (2018). In particular, the identification and quantification of these compounds was performed using a Thermo Ultimate™ 3000 HPLC (Thermo Scientific, Sunnyvale, CA, USA) coupled to a hybrid quadrupole‐orbitrap mass spectrometer (Q‐ExactiveTM; Thermo Scientific, Bremen, Germany), equipped with heated electrospray ionization (HESI‐II). Chromatographic separation was carried out by injecting 10 μL of sample on an Accucore™ Polar Premium LC column (150 mm × 3 mm, 2.6 μm particle size; Thermo Fisher Scientific, Waltham, MA, USA), using a water–acetonitrile gradient at a flow rate of 0.3 mL min^−1^. Mass detection was performed in negative ion mode using full MS data‐dependent MS/MS analysis (full MS–dd MS/MS). Full mass spectra were recorded with a resolution of 70,000 full width at half‐maximum (FWHM, calculated for *m*/*z* 200, 1.5 Hz), an automatic gain control (AGC) target of 5·10^5^ ions and a maximum injection time (IT) of 150 ms. Data‐dependent mass spectra were recorded with a resolution of 17,500 FWHM (*m*/*z* 200, 12 Hz), an AGC target of 1·10^5^ ions and an IT of 50 ms. The mass spectrometer was operated using the following parameters: spray voltage, 2.80 kV; sheath gas flow rate, 30 arbitrary units; auxiliary gas flow rate, 20 arbitrary units; capillary temperature, 310°C; capillary gas heater temperature, 280°C. Data acquisition and processing were carried out with Thermo Scientific™ Dionex™ Chromeleon™ 7.2 Chromatography Data System software.

### 2.5 Method validation

The characteristics of the method were studied using the 13 pure standards corresponding to the phenolic compounds quantified in the analyzed samples (acetovanillone, benzoic acid, coniferyl alcohol, coniferaldehyde, ferulic acid, homovanillic acid, methyl vanillate, 4‐hydroxybenzoic acid, *p*‐coumaric acid, pyrocatechol, scopoletin, vanillic acid and vanillin). Limits of quantification (LOQs) were established as 10 standard deviations of 10 replicated blank samples according to EURACHEM (1993). Method accuracy was estimated as recovery (%) of one sample spiked at two increasing concentration levels, covering the quantitation range of each phenol (high concentration, 1 mg L^−1^; low concentration, 0.2 mg L^−1^), each one analytically replicated three times. Precision was assessed as the relative standard deviation (RSD%) of the same experimental samples used for calculating accuracy.

### 2.6 Statistical analysis

Statistical analysis was performed using XLSTAT (version 2020, Addinsoft, France). Significant differences between the concentrations of phenolic compounds measured during different times of degradation were determined using the Kruskal–Wallis test (*p* < 0.05). Principal component analysis (PCA) was carried out to evaluate the relationships between phenolic compounds at different times of silver fir wood degradation and the activities of two fungal species.

## 3 RESULT AND DISCUSSION

### 3.1 Evaluation of sample preparation

The evaluation of the best conditions for sample preparation was conducted on a sample of silver fir sapwood taken from a living tree located in the Trentino Region (Italy). The use of different temperatures (30°C and 80°C) to dry the macerated wood samples did not influence the final content of phenolic compounds in the extracted samples (less than ±5%, data not shown). However, several studies showed that the use of high temperatures in the drying process might promote possible concurrent degradation of phenolic compounds.^67^, ^68^ For this reason, all samples were dried in the oven at 30°C. As regards the solvent mixtures, combinations of different solvents produced different recoveries of phenolic compounds, and the best compromise was obtained using H_2_O/MeOH at 75:25 v/v (Table 2).

**TABLE 2:** Phenolic contents (mg kg^−1^) extracted in silver fir sawdust samples with five solvent mixtures of methanol/water at 30°C

	Solvent mixture				
Compound	100% H_2_O	H_2_O/MeOH (75:25 v/v)	H_2_O/MeOH (50:50 v/v)	H_2_O/MeOH (25:75 v/v)	100% MeOH
Acetovanillone	0.020 ± 0.030	1.4 ± 0.060	0.100 ± 0.050	0.020 ± 0.020	0.010 ± 0.010
Benzoic acid	1.20 ± 0.218	1.42 ± 0.505	0.008 ± 0.003	0.658 ± 0.931	0.559 ± 0.788
Coniferyl alcohol	1.16 ± 0.153	0.431 ± 0.007	0.026 ± 0.008	0.008 ± 0.001	0.014 ± 0.006
Coniferaldehyde	0.065 ± 0.013	0.228 ± 0.303	0.030 ± 0.006	0.099 ± 0.001	0.044 ± 0.034
Ferulic acid	6.70 ± 2.40	8.60 ± 0.900	1.60 ± 0.100	1.70 ± 2.40	0.900 ± 0.600
Homovanillic acid	0.200 ± 0.100	4.30 ± 0.300	0.800 ± 0.500	1.10 ± 0.100	0.300 ± 0.100
Methyl vanillate	0.140 ± 0.085	0.460 ± 0.113	0.080 ± 0.028	0.140 ± 0.057	0.320 ± 0.198
4‐Hydroxybenzoic acid	7.30 ± 2.90	7.90 ± 4.10	4.00 ± 0.200	0.100 ± 0.100	0.100 ± 0.100
*p*‐Coumaric acid	2.50 ± 0.900	4.40 ± 0.500	0.400 ± 0.400	0.200 ± 0.020	0.100 ± 0.010
Pyrocatechol	0.016 ± 0.002	0.046 ± 0.059	0.005 ± 0.001	0.012 ± 0.001	0.005 ± 0.001
Scopoletin	0.100 ± 0.141	0.200 ± 0.100	0.100 ± 0.141	0.100 ± 0.141	0.200 ± 0.100
Vanillic acid	0.500 ± 0.020	12.9 ± 8.20	2.90 ± 0.100	0.400 ± 0.600	0.100 ± 0.100
Vanillin	5.00 ± 1.00	30.0 ± 7.30	10.0 ± 1.00	23.0 ± 3.00	20.6 ± 3.40

Note: Values are average ± standard deviation of triplicate analysis.

### 3.2 Method validation

**FIGURE 1:**
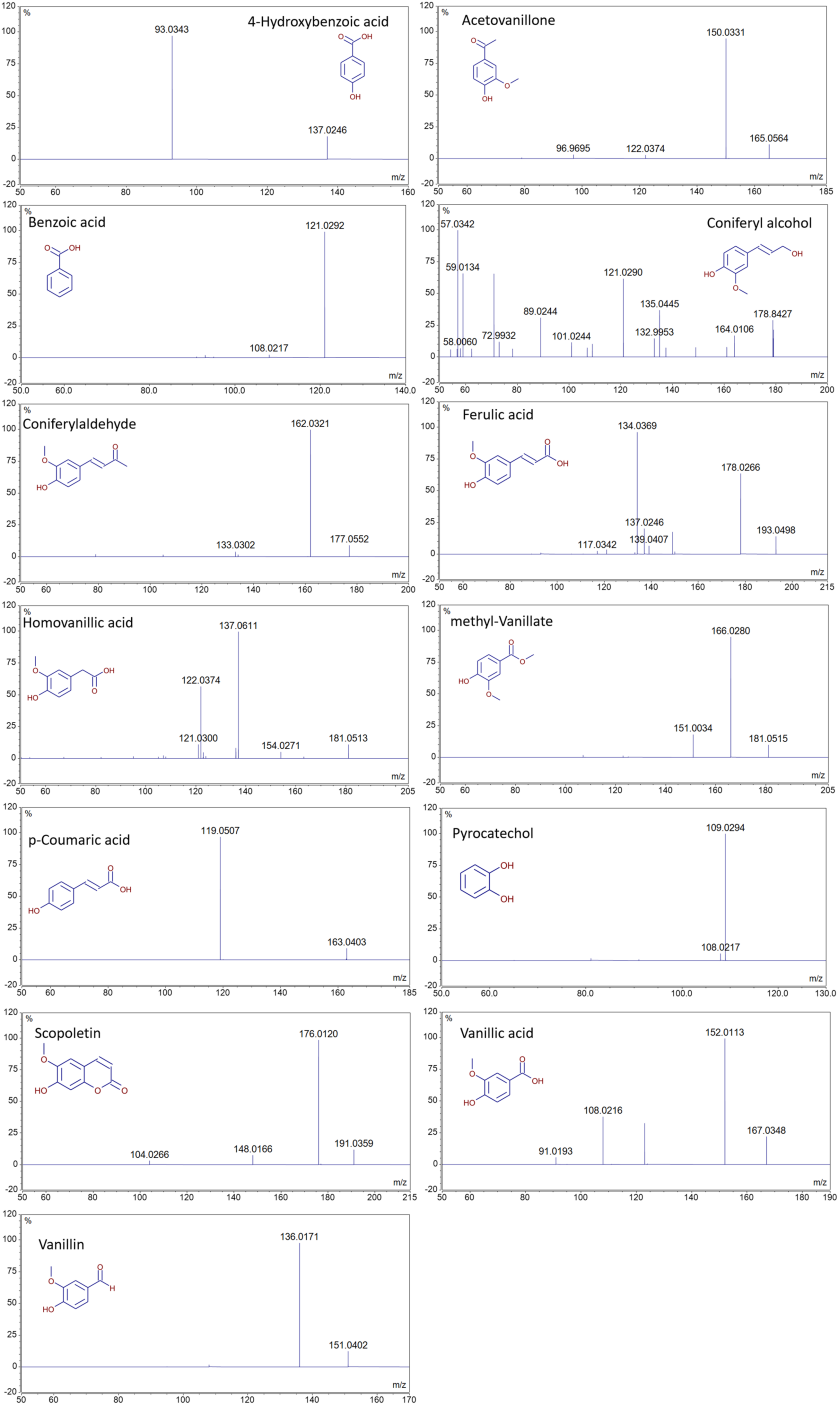
Fragmentation spectra (ddscan MS/MS) of detected phenols

Phenol quantification was performed on precursor ions detected in the extracted ion chromatograms (EICs) corresponding to the deprotonated molecules [M‐H]^−^. Due to confirmed sample compounds, accuracy‐mass tolerance was set at <5 ppm and RT and dd‐MS/MS spectra were compared with those collected from available standards (Figure 1).

**FIGURE 2:**
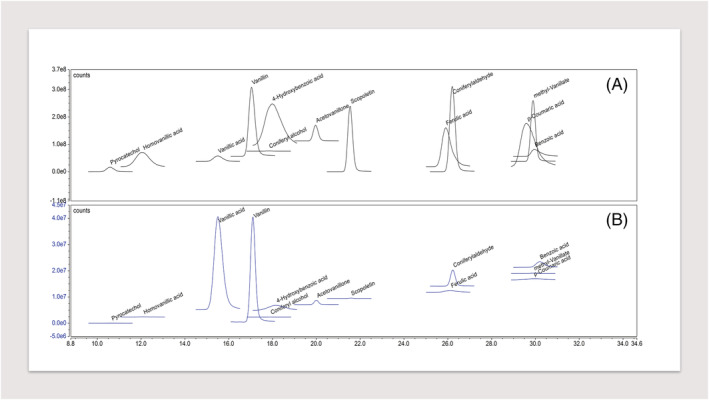
Chromatograms of (a) standard phenol mixture solution at 2 mg/L and (b) wood sample

The method characteristics including LOQ, linearity range, precision and accuracy determined for each phenolic compound are shown in Table 3. Accuracy, evaluated in terms of recovery (%), was between 40% and 77% in the samples supplemented with 0.2 mg L^−1^ of standard mixture solution, while it was between 51% and 106% in the samples supplemented with 1 mg L^−1^ of standard mixture solution. The precision values (expressed as relative standard deviations; RSD %) were always below 8% for the samples supplemented with 1 mg/L of standard mixture solution, while for those supplemented with 0.2 mg L^−1^ the values were always lower than 16% (Table 3). The MS quantification channel chromatograms of a standard mixture solution and a sample are reported in Figure 2.

**TABLE 3:** Validation parameters of 13 phenolic compounds detected in silver fir sawdust samples for LC‐Q‐Orbitrap analysis

				LOQ	Linearity range	Precision (RSD %)		Accuracy (%)	
Phenolic compound	RT (min)	[M‐H]^−^ (*m*/*z*)	Fragment (*m*/*z*)	μg mL^−1^		0.2	1	0.2	1
						mg/L^−1^			
**Simple phenols**									
Pyrocatechol	10.6	109.0295	108.022	0.0005	0.0005–8.95	16	2	71	82
**Alkyphenyl alcohols**									
Coniferyl alcohol	17.8	179.0714	121.029	0.0107	0.0107–5.35	8	8	43	51
**Hydroxybenzoketones**									
Acetovanillone	20.0	165.0557	150.033	0.0001	0.0001–5.12	12	2	45	76
Methyl vanillate	29.9	181.0506	166.028	0.0005	0.0005–9.27	8	2	59	69
**Hydroxycinnamaldehydes**									
Coniferylaldehyde	26.2	177.0556	162.032	0.0001	0.0001–5.07	2	4	43	51
**Hydroxybenzaldehydes**									
Vanillin	17.1	151.0401	136.017	0.0001	0.0001–5.36	3	6	53	71
**Hydroxyphenylacetic acids**									
Homovanillic acid	12.1	181.0506	137.061	0.0010	0.0010–2.97	8	4	52	79
**Hydroxycinnamic acids**									
Ferulic acid	26.0	193.0506	134.037	0.0001	0.0001–6.21	5	3	47	51
*p*‐Coumaric acid	29.6	163.0401	119.051	0.0001	0.0001–5.20	7	4	41	74
**Hydroxybenzoic acids**									
Benzoic acid	30.0	121.0295	108.022	0.001	0.001–5	10	1	49	90
4‐Hydroxybenzoic acid	18.1	137.0244	93.034	0.0001	0.0001–5.28	7	3	67	106
Vanillic acid	15.5	167.0350	152.011	0.0001	0.0001–3.04	10	2	77	58
**Hydroxycoumarins**									
Scopoletin	21.5	191.0350	176.012	0.0010	0.0010–9.11	11	3	40	60

Note: RT = retention time; LOQ = limit of quantification.*Linearity ranges and LOQs are defined without considering sample dilution.

### 3.3 Phenolic compounds in silver fir wood

Several phenolic compounds were identified in silver fir wood samples. As reported in Table S1, among simple phenols, a low content of pyrocatechol (from 0.29 to 0.44 mg kg^−1^) was detected. To the best of our knowledge, this compound was found for the first time in fresh silver fir wood, while other studies showed the production of pyrocatechol due to the action of ligninolytic enzymes of white rot fungi during lignin degradation.^69^, ^70^ As regards alkylphenyl alcohols, a high concentration of coniferyl alcohol (from 5.9 to 13.9 mg kg^−1^) was found. Coniferyl alcohol is one of the main components of lignin, particularly abundant in coniferous species, such as silver fir.^71^ Among hydroxybenzaldehydes, notably high concentrations of vanillin (from 39.4 to 58.7 mg kg^−1^) were found. These results agree with Zarzyński,^72^ concerning the identification and quantification of phenolic compounds in wood of exotic and European tree species, such as silver fir. As regards hydroxybenzoketones, acetovanillone and methyl vanillate were found in detectable amounts, with concentrations ranging from 2.5 to 3.9 mg kg^−1^ and from 0.15 to 0.20 mg kg^−1^, respectively. These compounds were detected for the first time in silver fir wood. Among hydroxycinnamic acids, a low content of ferulic acid (from 0.05 to 0.50 mg kg^−1^) was found. This compound was previously identified in wood of other coniferous species, such as Norway spruce, by Metsämuuronen and Siren (2014). Finally, as regards hydroxybenzoic acids, benzoic acid, 4‐hydroxybenzoic acid and vanillic acids were found with remarkable concentrations ranging from 14.2 to 22.8 mg kg^−1^, from 4.4 to 6.3 mg kg^−1^ and from 59.3 to 79.7 mg kg^−1^, respectively.

In particular, among all the phenolic compounds, vanillic acid was the most abundant. Few studies reported the presence of this compound in the bark and wood extract of silver fir.^73^, ^74^

### 3.4 Effect of fungal activity on phenolic components

**FIGURE 3:**
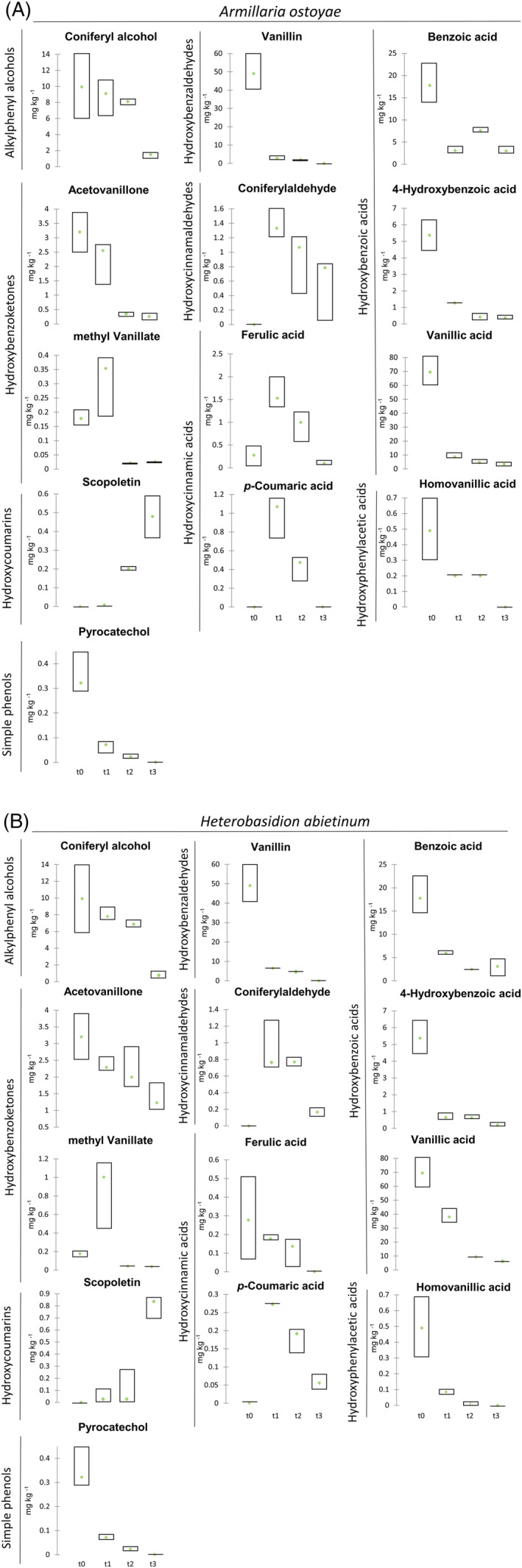
(a, b) Box plots with phenolic compound content (mg kg^−1^ and μg kg^−1^ for isoacetosyringone) at times t0, t1, t2 and t3 (different times of macerated silver fir wood degradation) by *Armillaria ostoyae* and *Heterobasidion abietinum*

The changes in the phenolic profiles of silver fir wood, in relation to the activity of the two different fungal species belonging to the *Armillaria* and *Heterobasidion* genera, were evaluated during 6 months of wood degradation. The phenol content was analyzed at 2, 4 and 6 months. Table S1 summarizes the phenolic compound content, while the trends of these compounds at different times of degradation can be observed in Figure 3(a, b).

Specifically, considering phenols already available in macerated wood, a similar decay pattern was observed for coniferyl alcohol, vanillin, benzoic acid, 4‐hydroxybenzoic acid and pyrocatechol for both rot fungi.

In particular, vanillin, benzoic acid, 4‐hydroxybenzoic acid and pyrocatechol were rapidly metabolized by both fungal species, showing a decreasing trend during the degradation process with a total consumption of vanillin and pyrocatechol. Considering vanillin, despite starting from high concentrations (about 50 mg kg^−1^), complete degradation of this compound was observed during the decay process, differently from what was reported by several studies that observed the production of vanillin during lignin degradation by different species of basidiomycete fungi like *Aspergillus sydowii*,^75^ *Pycnoporus cinnabarinus* ^76^ and *Schizophyllum commune*.^77^ Moreover, in some fungal species vanillin was described as an intermediate during lignin degradation for the conversion of ferulic acid to vanillic acid.^75^, ^77^ Benzoic acid and 4‐hydroxybenzoic acid were already identified as lignin degradation products,^78^, ^79^, ^80^ and several studies reported that benzoic might arise after the oxidative cleavage of the α and β carbons of the alkyl side chain by MnP.^31^, ^81^ As regards pyrocatechol, studies conducted on the white rot fungus *P. chrysosporium* highlighted the production of this compound due to the oxidation of β‐O‐4 linkages of lignin by lignin peroxidase.^69^, ^70^ However, other studies indicated a decrease of pyrocatechol levels after the action of laccase with the subsequent conversion to phenoxyl radicals through oxidation processes.^19^, ^82^

Regarding coniferyl alcohol, the decreasing trend during the degradation process appeared much slower for both fungi and the consumption of phenols was not complete. Coniferyl alcohol showed a concentration significantly lower after 6 months (t3) from fungal inoculation compared to the other time points (Table S1). This alcohol is reported as one of the most abundant monolignols of softwood lignin polymer^23^ and several studies described its degradation by fungi and bacteria with the consequent production of ferulic acid, coniferylaldehyde and vanillic acid.^76^, ^83^, ^84^

Among the other phenols present in wood at t0, acetovanillone levels showed a decreasing trend for both fungal species, but for *A. ostoyae* the consumption of this phenol was faster and almost complete already after 4 months. As regards acetovanillone, several studies reported the presence of this compound during lignin degradation by white rot fungi.^85^, ^86^ A significantly decrease of ferulic acid levels was observed for *H. abietinum*, while an initial accumulation and subsequent degradation appeared in the samples inoculated with *A. ostoyae*. In the literature, different pathways of ferulic acid degradation induced by different fungal species were reported^76^, ^87^, ^88^ with the formation of other phenolic compounds, such as vanillic acid and coniferylaldehyde.^76^ A similar behavior with an initial accumulation (t1) was observed for methyl vanillate, which presented a low initial concentration in wood (t0), especially in wood samples inoculated with *H. abietinum*. However, in the following months, methyl vanillate was completely consumed (Figure 3).

Homovanillic acid was completely consumed. The consumption was very fast for *H. abietinum* and more gradual for *A. ostoyae*. On the contrary, Takada et al.^89^ reported that homovanillic acid was produced after lignin degradation by fungi. Vanillic acid levels showed a decreasing trend during degradation by both fungal species, but was completely consumed only in samples degraded by *H. abietinum*. In wood samples degraded by *A. ostoyae*, the degradation was faster but not complete.

During wood degradation, the formation of coniferaldehyde, *p*‐coumaric acid and scopoletin was observed; these compounds were initially totally absent in the macerated silver fir wood. At 2 months (t1) after the inoculation of both fungal species, the coniferylaldehyde concentration exceeded 1 mg kg^−1^ and subsequently decreased until this compound was almost completely degraded at 6 months (t3). Several studies reported the production of this compound after the enzymatic degradation of coniferyl alcohol by white rot fungi.^84^, ^90^ Moreover, Falconnier et al.^76^ observed the formation of coniferylaldehyde after the reduction of the propenoic side chain of ferulic acid by the action of ligninolytic enzymes in the white rot fungus *Trametes versicolor*. Several studies reported the presence of *p*‐coumaric acid due to the action of lignin‐degrading enzymes.^56^, ^91^ In particular, in both species there was an initial accumulation, with average contents of about 0.99 mg kg^−1^ and 0.27 mg kg^−1^ of this phenol after 2 months (t1) with *A. ostoyae* and *H. abietinum*, respectively. After that, degradation was observed in both cases, with total consumption only in the samples inoculated with *A. ostoyae*. Several studies reported different strategies of *p*‐coumaric acid degradation by various fungal species.^92^, ^93^, ^94^ For example, in the basidiomycete *P. cinnabarinus*, the oxidative degradation of the *p*‐coumaric acid side chain led to the formation of *p*‐hydroxybenzoic acid.^92^

Finally, the hydroxycoumarin scopoletin was the only phenol investigated that showed accumulation during degradation, reaching a concentration of 0.6 and 0.9 mg kg^−1^ after 6 months (t3) in samples inoculated with *A. ostoyae* and *H. abietinum*, respectively. Scopoletin is produced after the degradation of ferulic acid via the phenylpropanoid pathway^95^ due to the action of ligninolytic enzymes, such as MnP, lignin peroxidase and laccase, secreted in both fungal species.^96^ Usually its accumulation is correlated with different kinds of stress, such as the resistance to microbial attack.^97^ Rodriguez et al.^98^ mentioned the possible antimicrobial functions of scopoletin against a variety of different fungal and bacterial infections.

The decreases observed for many of the detected phenolic compounds indicated that different fungal species are able to efficiently metabolize most of the phenolic compounds.^99^ Despite the diversity of enzymatic strategies of lignin depolymerization by the two different fungal species belonging to the *Armillaria* and *Heterobasidion* genera, the trends of phenolic compounds were not so variable. However, according to previous studies,^24^, ^100^ the degradation rates and the final concentrations at t3 are significantly different for acetovanillone, coniferaldehyde, ferulic acid and homovanillic acid (Kruskal–Wallis test, *P* < 0.05; Table S1).

### 3.5 Phenolic profiles and degradative fungal activity

**FIGURE 4:**
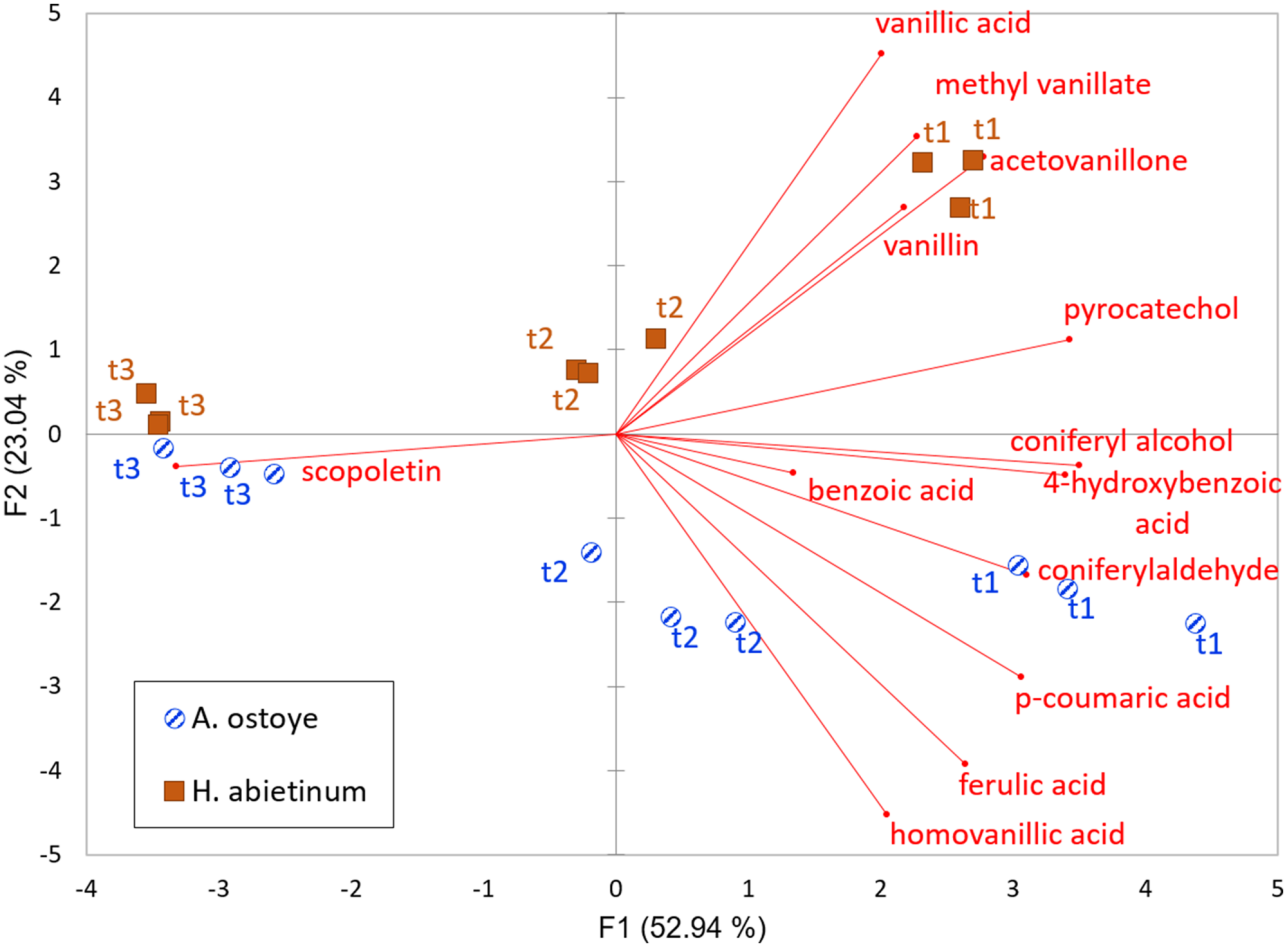
Principal component analysis (PCA) with the distribution of phenolic compounds at times t0, t1, t2 and t3 (different times of macerated silver fir wood degradation) by *Armillaria ostoyae* and *Heterobasidion abietinum*

PCA was applied for the content of each phenolic compound quantified in macerated silver fir wood samples, in order to evaluate the correlations between phenolic profiles and the activities of two fungal species at different times of wood degradation (Figure 4). PCA, with PC1 and PC2 collectively accounting for 76% of total variance, revealed a good differentiation between the activities of the two fungal species during the first period (at t1 and t2) of silver fir wood degradation. In particular, PC1 clearly separates the data based on time since inoculation and PC2 separates the data based on the fungal species. Vanillic acid, methyl vanillate, acetovanillone and vanillin better explained samples inoculated with *H. abietinum* after 2 months of degradation, while, at the same time, coniferaldehyde and *p*‐coumaric acid better explained samples inoculated with *A. ostoyae*. In particular, vanillin and vanillic acid were already present in wood before the degradation process; these two compounds were probably released from the degradation of guaiacyl‐β‐coniferyl ether.^37^ Subsequently, they underwent rapid consumption, while the methyl vanillate concentration increased at t1. It was hypothesized that this formation could directly be derived from the methylation of vanillic acid by the fungi. Considering the greater production of methyl vanillate in wood samples degraded by *H. abietinum*, but at the same time a lower consumption of vanillic acid and a higher consumption of ferulic acid, *H. abietinum* might converted ferulic acid to vanillic acid, as reported for the ubiquitous white rot fungus *S. commune*.^101^ For what concerns coniferaldehyde and *p*‐coumaric acid, both were produced due to the action of lignin‐degrading enzymes,^37^, ^56^ with a particularly quicker production for *p*‐coumaric by *H. abietinum*.

Subsequently (t3, 6 months), a homogenization was observed of the samples based on the degradation of various investigated phenols and the accumulation of scopoletin in samples inoculated with both fungal species, suggesting a possible use of this parameter as a marker indicative of fungal degradation of wood.

## Supporting information


**TABLE S1:** Statistical distribution of phenolic content detected in silver fir sawdust samples at time t0 and at different times of silver fir wood degradation (t1, t2 and t3) by *Armillaria ostoyae* and *Heterobasidion abietinum*. Data are expressed in mg kg^−1^ for all phenolic compounds.

## Data Availability

Research data are not shared
