# Supplementary material for: White rot fungal impact on the evolution of simple phenols during decay of silver fir wood by UHPLC‐HQOMS
Source: Phytochem Anal. 2021 Jul 28;33(2):170–83. doi: 10.1002/pca.3077 (PMC9290616; doi:10.1002/pca.3077)
Supplement: Supplementary file 1 — TABLE S1: Statistical distribution of phenolic content detected in silver fir sawdust samples at time t0 and at different times of silver fir wood degradation (t1, t2 and t3) by Armillaria ostoyae and Heterobasidion abietinum. Data are expressed in mg kg−1 for all phenolic compounds. [file PCA-33-170-s001.docx]

**TABLE 1S**

Statistical distribution of phenolic content detected in silver fir sawdust samples at time (t0) and during different times of silver fir wood degradation (t1, t2 and t3) by *Armillaria ostoyae* and *Heterobasidion* *abietinum*. Data is expressed in mg kg^-1^ for all phenolic compounds.

| **Compounds** | **time** | ***Armillaria ostoyae*** | | | ***Heterobasidion abietinum*** | | |
| --- | --- | --- | --- | --- | --- | --- | --- |
|  |  | **Min** | **Mdn** | **Max** | **Min** | **Mdn** | **Max** |
| **Simple phenols** |  |  |  |  |  |  |  |
| Pyrocatechol | t0 | 0.288 | 0.322**^a^** | 0.436 | 0.288 | 0.322**^a^** | 0.436 |
|  | t1 | 0.042 | 0.072**^ab^** | 0.084 | 0.060 | 0.070**^ab^** | 0.088 |
|  | t2 | 0.016 | 0.023**^ab^** | 0.030 | 0.012 | 0.015**^ab^** | 0.018 |
|  | t3 | <LOQ | <LOQ**^b^** | <LOQ | <LOQ | <LOQ**^b^** | <LOQ |
| **Alkylphenyl alcohols** | | | | | | | |
| Coniferyl alcohol | t0 | 5.94 | 9.93**^a^** | 13.9 | 5.94 | 9.93**^a^** | 13.9 |
|  | t1 | 6.35 | 9.11**^a^** | 10.8 | 7.53 | 7.61**^a^** | 8.89 |
|  | t2 | 7.74 | 8.05**^a^** | 8.36 | 6.43 | 6.95**^a^** | 7.27 |
|  | t3 | 0.726 | 1.37**^b^** | 1.46 | 0.406 | 0.724**^b^** | 0.814 |
| **Hydroxybenzoketones** |  |  |  |  |  |  |  |
| Acetovanillone | t0 | 2.51 | 3.20**^a^** | 3.89 | 2.51 | 3.20**^a^** | 3.89 |
|  | t1 | 1.36 | 2.56**^ab^** | 2.76 | 2.13 | 2.29**^ab^** | 2.57 |
|  | t2 | 0.302 | 0.348**^ab^** | 0.364 | 1.64 | 2.00**^ab^** | 2.86 |
|  | t3 | <LOQ | 0.212**^b^** | 0.260 | 0.964 | 1.23**^b^** | 1.74 |
| Methyl vanillate | t0 | 0.152 | 0.178**^a^** | 0.204 | 0.152 | 0.178**^ab^** | 0.204 |
|  | t1 | 0.180 | 0.354**^a^** | 0.382 | 0.474 | 1.00**^a^** | 1.16 |
|  | t2 | 0.022 | 0.028**^a^** | 0.028 | 0.030 | 0.030**^b^** | 0.044 |
|  | t3 | 0.022 | 0.026**^a^** | 0.030 | 0.034 | 0.034**^ab^** | 0.038 |
| **Hydroxycinnamaldehydes** | |  |  |  |  |  |  |
| Coniferylaldehyde | t0 | <LOQ | <LOQ**^b^** | <LOQ | <LOQ | <LOQ**^b^** | <LOQ |
|  | t1 | 1.20 | 1.33**^a^** | 1.60 | 0.708 | 0.730**^ab^** | 1.28 |
|  | t2 | 0.424 | 1.07**^ab^** | 1.20 | 0.756 | 0.770**^ab^** | 0.854 |
|  | t3 | 0.050 | 0.832**^ab^** | 0.840 | 0.096 | 0.166**^a^** | 0.184 |
| **Hydroxybenzaldehydes** |  |  |  |  |  |  |  |
| Vanillin | t0 | 39.4 | 49.1**^a^** | 58.7 | 39.4 | 49.1**^a^** | 58.7 |
|  | t1 | 2.11 | 2.73**^ab^** | 3.31 | 34.3 | 38.0**^ab^** | 42.8 |
|  | t2 | 1.95 | 1.98**^ab^** | 2.01 | 8.19 | 8.70**^ab^** | 9.42 |
|  | t3 | <LOQ | <LOQ**^b^** | <LOQ | 5.96 | 6.13**^b^** | 6.48 |
| **Hydroxyphenylacetic acids** | | | | | | | |
| Homovanillic acid | t0 | 0.298 | 0.490**^a^** | 0.682 | 0.298 | 0.490**^a^** | 0.682 |
|  | t1 | 0.126 | 0.200**^ab^** | 0.252 | 0.050 | 0.100**^b^** | 0.123 |
|  | t2 | 0.114 | 0.141**^ab^** | 0.168 | 0.028 | 0.034**^b^** | 0.062 |
|  | t3 | <LOQ | <LOQ**^b^** | <LOQ | <LOQ | <LOQ**^b^** | <LOQ |
| **Hydroxycinammic acids** | | | | | | | |
| Ferulic acid | t0 | 0.050 | 0.277**^ab^** | 0.504 | 0.050 | 0.277**^a^** | 0.504 |
|  | t1 | 1.44 | 1.53**^a^** | 2.09 | 0.160 | 0.172**^a^** | 0.178 |
|  | t2 | 0.490 | 0.998**^ab^** | 1.13 | 0.014 | 0.136**^a^** | 0.164 |
|  | t3 | 0.052 | 0.054**^b^** | 0.094 | <LOQ | <LOQ**^a^** | <LOQ |
| *p*-Coumaric acid | t0 | <LOQ | <LOQ**^b^** | <LOQ | <LOQ | <LOQ**^b^** | <LOQ |
|  | t1 | 0.748 | 1.07**^a^** | 1.16 | 0.272 | 0.273**^a^** | 0.274 |
|  | t2 | 0.288 | 0.476**^ab^** | 0.526 | 0.138 | 0.196**^ab^** | 0.202 |
|  | t3 | <LOQ | <LOQ**^b^** | <LOQ | 0.050 | 0.056**^ab^** | 0.088 |
| **Hydroxybenzoic acids** |  |  |  |  |  |  |  |
| Benzoic acid | t0 | 14.2 | 17.8**^a^** | 22.8 | 14.2 | 17.8**^a^** | 22.8 |
|  | t1 | 2.54 | 3.11**^a^** | 3.92 | 4.96 | 5.03**^ab^** | 5.93 |
|  | t2 | 6.51 | 6.61**^a^** | 7.57 | 2.48 | 2.50**^b^** | 2.51 |
|  | t3 | 2.47 | 2.58**^a^** | 4.21 | 3.09 | 3.44**^ab^** | 3.79 |
| 4-hydroxybenzoic acid | t0 | 4.41 | 5.37**^a^** | 6.33 | 4.41 | 5.37**^a^** | 6.33 |
|  | t1 | 1.24 | 1.26**^ab^** | 1.28 | 0.648 | 0.710**^ab^** | 0.956 |
|  | t2 | 0.218 | 0.415**^ab^** | 0.612 | 0.574 | 0.636**^ab^** | 0.698 |
|  | t3 | 0.214 | 0.287**^b^** | 0.360 | 0.144 | 0.206**^b^** | 0.244 |
| Vanillic acid | t0 | 59.3 | 69.5**^a^** | 79.7 | 59.3 | 69.5**^a^** | 79.7 |
|  | t1 | 7.28 | 8.74**^ab^** | 10.2 | 34.3 | 38.0**^ab^** | 42.8 |
|  | t2 | 4.50 | 4.76**^ab^** | 5.88 | 8.19 | 8.70**^ab^** | 9.42 |
|  | t3 | 2.07 | 3.09**^b^** | 3.27 | 5.96 | 6.22**^b^** | 6.48 |
| **Hydroxycoumarins** | | | | | | | |
| Scopoletin | t0 | <LOQ | <LOQ**^b^** | <LOQ | <LOQ | <LOQ**^b^** | <LOQ |
|  | t1 | 0.004 | 0.004**^ab^** | 0.008 | <LOQ | 0.008**^ab^** | 0.110 |
|  | t2 | 0.200 | 0.202**^ab^** | 0.210 | 0.012 | 0.012**^ab^** | 0.278 |
|  | t3 | 0.368 | 0.480**^a^** | 0.592 | 0.700 | 0.872**^a^** | 0.872 |

Note: Min = minimum content; Mdn = median content; Max = maximum content; LOQ= limit of quantification; a, b = significant differences, Kruskal-Wallis test (p <0.05).
